# Supplementary material for: Lead transfer in the soil-root-plant system in a highly contaminated Andean area
Source: PeerJ. 2021 Jan 5;9:e10624. doi: 10.7717/peerj.10624 (PMC7792523; doi:10.7717/peerj.10624)
Supplement: Supplemental Information 1 [file peerj-09-10624-s001.pdf]

Raw data:

Concentration, transfer and bioaccumulation of lead in the soil-root-sprout system in natural grass and cultivated in rainy and dry seasons - 2018

Season	Grass type	Pb in soil (mg(kg)	Pb in root (mg/kg)	Pb in shoot (mg/kg)	Soil-root BCF	Root-shoot TF	Soil-shoot BF
1	1	234.17	229.31	21.52	0.98	0.09	0.09
1	1	251.22	127.10	20.16	0.51	0.16	0.08
1	1	264.92	137.06	23.46	0.52	0.17	0.09
1	1	189.85	102.73	19.36	0.54	0.19	0.10
1	1	244.67	263.61	18.36	1.08	0.07	0.08
1	1	284.13	208.25	21.51	0.73	0.10	0.08
1	1	237.64	128.24	16.65	0.54	0.13	0.07
1	1	174.51	117.28	18.58	0.67	0.16	0.11
1	1	171.12	140.48	23.88	0.82	0.17	0.14
1	1	195.25	237.28	23.77	1.22	0.10	0.12
1	2	264.92	203.39	23.81	0.77	0.12	0.09
1	2	131.76	153.58	19.24	1.17	0.13	0.15
1	2	175.27	251.49	17.56	1.43	0.07	0.10
1	2	234.17	221.25	19.56	0.94	0.09	0.08
1	2	198.26	102.92	22.23	0.52	0.22	0.11
1	2	225.65	114.68	18.78	0.51	0.16	0.08
1	2	256.65	171.39	15.22	0.67	0.09	0.06
1	2	198.76	154.49	23.17	0.78	0.15	0.12
1	2	188.69	229.72	14.55	1.22	0.06	0.08
1	2	234.60	240.74	20.55	1.03	0.09	0.09
2	1	222.47	197.72	20.45	0.89	0.10	0.09
2	1	238.64	77.04	19.14	0.32	0.25	0.08
2	1	251.65	102.75	22.30	0.41	0.22	0.09
2	1	180.37	95.34	18.40	0.53	0.19	0.10
2	1	232.43	171.99	17.44	0.74	0.10	0.08
2	1	269.93	156.17	20.42	0.58	0.13	0.08
2	1	225.75	96.18	15.81	0.43	0.16	0.07
2	1	165.79	87.95	17.64	0.53	0.20	0.11
2	1	162.54	105.34	22.68	0.65	0.22	0.14
2	1	185.44	172.32	22.59	0.93	0.13	0.12
2	2	251.68	77.21	22.64	0.31	0.29	0.09
2	2	125.18	86.04	22.60	0.69	0.26	0.18
2	2	166.53	128.56	16.70	0.77	0.13	0.10
2	2	222.44	115.86	18.59	0.52	0.16	0.08
2	2	188.33	165.93	21.22	0.88	0.13	0.11

2	2	214.38	177.94	17.84	0.83	0.10	0.08
2	2	243.81	152.53	14.47	0.63	0.09	0.06
2	2	188.83	115.17	22.02	0.61	0.19	0.12
2	2	179.21	180.52	13.83	1.01	0.08	0.08
2	2	222.84	188.63	19.53	0.85	0.10	0.09

1	Rainy season
2	Dry season

1	Natural pasture
2	Cultivated pasture

BCF: bioconcentration factor
TF: translocation factor
BF: bioaccumulation factor
